# Adjusted Rand Index-Guided DPSO for Clustering and Data Routing in Wireless Sensor Networks

**DOI:** 10.3390/s26123700

**Published:** 2026-06-10

**Authors:** Sidi Mohamed Mohi Dine, Zhiyi Zhu, Patrick Finnerty, Chikara Ohta

**Affiliations:** Graduate School of System Informatics, Kobe University, Kobe 657-0013, Japan; 194x606x@gsuite.kobe-u.ac.jp (Z.Z.); finnerty.patrick@boar.kobe-u.ac.jp (P.F.); ohta@port.kobe-u.ac.jp (C.O.)

**Keywords:** wireless sensor networks, network lifetime maximization, clustering algorithms, energy efficient routing, discrete particle swarm optimization, adjusted Rand index, adaptive perturbation

## Abstract

Establishing an energy-balanced data routing and clustering approach is among the most fundamental steps to extend the longevity of wireless sensor networks (WSNs). This study presents an intelligent and energy-aware framework for data routing and clustering in WSN employing an adjusted Rand index (ARI)-guided discrete particle swarm optimization algorithm: ARI-DPSO. This method uses Dijkstra’s algorithm to establish energy-efficient data paths and uses the network lifetime as the ARI-DPSO’s fitness function. The discrete particle swarm optimization searches for the globally optimal cluster configuration that extends the network’s operational lifetime. The novelty of the ARI-DPSO lies in its capability to avoid premature convergence by using the ARI metric to quantify the similarity between the swarm’s global best solution and the current particles. Based on this level of similarity, ARI-DPSO employs an ARI-derived mechanism to trigger a dynamic perturbation element to force the swarm of particles to explore new search areas. The simulation results show that the ARI-DPSO improves the dynamics and diversity of the swarm, thereby maximizing the stable operational lifetime of wireless sensor networks (first node death).

## 1. Introduction

Wireless sensor networks have played an essential role in the rapid evolution of the Internet of Things (IoT), enabling its use in various industrial and environmental applications [1]. The limited energy supply poses a critical constraint on the viability of these tiny sensors, making maximizing their lifetimes a key design challenge [2]. Hierarchical clustering and data routing protocols have demonstrated significant efficiency in mitigating the primary source of energy consumption, namely the energy used for data aggregation and transmission across the network [3]. Clustering is a technique that partitions sensor networks into localized clusters, with each cluster containing a designated node as the cluster head (CH) to collect, aggregate, and transmit data from the cluster members to the base station [4]. Obtaining the optimal node-to-cluster configuration is computationally expensive for dense wireless networks, as it involves considering a tremendously large number of node assignments due to the combinatorial explosion of the solution space [5].

While deterministic clustering and routing methods, such as chain- and tree-based protocols [6,7], offer locally efficient solutions, they may not converge to a globally optimal solution. Advanced metaheuristic algorithms provide optimal solutions within reasonable computational resources [8] through a stochastic, intelligent, and self-correcting search framework. Adopting PSO techniques to optimize node-to-cluster membership requires a mathematical compatibility to apply continuous PSO search to a discrete search space [9]. Since the canonical PSO was introduced to search for an optimal solution in a continuous search space, it struggles with the combinatorial nature of the discrete space [10]. The discretization of PSO forces the particles to get trapped in the local minima, leading to premature convergence and diminishing the swarm diversity [11].

The discrete nature of applications, such as node-to-cluster assignment, imposes strict connectivity and topological constraints. Aggressively updating the particle search velocity may violate these constraints and lead to situations where particles contain clusters that are not connected or cannot transmit to the next destination. However, timid updates result in mediocre diversity, which may lead the swarm to get trapped in the local minima and trigger premature convergence. Therefore, it is critical to equip the discrete PSO with an adaptability feature that enables it to simultaneously escape premature convergence without violating network constraints.

The adjusted Rand index (ARI) is a robust metric that quantifies the similarity between two partitions by examining the pairwise assignments of members in both partitions [12]. In the context of WSNs, pairwise member assignment means that the ARI checks whether each pair of nodes is grouped in the same cluster under both clustering configurations, or whether the pair is split between two distinct clusters. Based on the pairwise check, the ARI yields a similarity score ranging from −1 to 1, where 1 indicates complete agreement between the partitions being compared, 0 implies a level of similarity expected by random chance, and negative values indicate that the level of similarity is worse than random chance.

To exploit the potential search capability of the discrete PSO while mitigating premature convergence, we employ the adjusted Rand index-guided discrete particle swarm optimization (ARI-DPSO) algorithm. The novelty of this study lies in enhancing the swarm’s diversity by improving its exploration capabilities. Specifically, we use the ARI metric to quantify the similarity between each particle’s cluster configuration and the swarm’s global best solution ($g_{\mathrm{best}}$). Then, we employ an ARI-derived mechanism to force individual particles to unlock new search areas, thereby enhancing the swarm’s search behavior.

Accordingly, the enhanced search behavior enables the swarm-optimization search to achieve energy-efficient cluster configuration and, consequently, extend the network lifetime. In the context of WSNs, network lifetime is defined by two distinct phases: the operational lifetime, defined as the lifetime until the last node death (LND), and the stable network lifetime, defined as the time until the first node death (FND). While LND measures the whole time taken until the death of the last node, FND is adopted as a lifetime definition for applications that require sensitive and accurate data from each sensor in the network. This study adopts FND as the main optimization metric. The fitness function of the ARI-DPSO is the lifetime obtained from the routing model used in this study, which employs Dijkstra’s algorithm to construct locally minimum-energy paths.

To the best of our knowledge, the ARI metric has been used primarily as a diagnostic tool to assess the similarity between the final clustering results of two or more approaches in the domain of WSN clustering [13,14]. The primary novelty of the ARI-DPSO is that it shifts the application of the ARI metric from a simple diagnostic tool to an algorithmic driver, using it as a detection mechanism to prevent premature convergence.

The contributions of this study are summarized as follows:

- **Novel adaptive mechanism:** We present a novel application of the ARI metric as a real-time stagnation detection mechanism by using this metric as an active tool to guide and control the swarm’s search.
- **Premature Convergence Regulation:** We employ an ARI-derived mechanism to enhance the swarm’s search behavior by dynamically forcing exploratory moves away from stagnation areas.
- **Extended network longevity**: Results from our extensive simulations reveal that the ARI-DPSO achieves a significant superiority over the genetic algorithm (GA), ant colony optimization (ACO), the standard DPSO, and the state-of-the-art energy-balanced path tree clustering and routing algorithm (EBPT-CRA) [15], in terms of extending the stable network lifetime (first node death).
- **Search behavior:** This study presents an in-depth search behavioral-diagnostic by tracking the swarm diversity and the history of the global best fitness. This quantification of behavior reveals the high diversity of the ARI-DPSO and its ongoing attempts to explore new search areas.

The rest of the paper is organized as follows: in Section 2, we present a review of some of the techniques used in data routing and clustering in WSNs. We detailed the methodology of the ARI-DPSO method in Section 3. In Section 4, we present a performance analysis of ARI-DPSO, and we compare it to the standard DPSO and the state-of-the-art EBPT-CRA. Finally, we summarize the findings of this study and highlight promising domains of application of this method in Section 5.

## 2. Related Work

### 2.1. Hybrid Cluster-Tree Clustering and Routing Methods

Low-energy adaptive clustering hierarchy (LEACH) is an adaptive routing protocol designed to reduce energy consumption in wireless sensor networks by grouping nodes into clusters [16]. A special node is chosen as the cluster head (CH) to collect data from the member nodes and transmit it to the sink. To ensure that the high-energy duty of the cluster head is rotated among all nodes in each cluster, LEACH employs a probabilistic approach to select the cluster head [17]. Mao et al. [18] improved the selection of the cluster head in LEACH by considering the distance to the sink and the residual energy of the cluster head candidates, while Bhih et al. [19] proposed the use of the LEACH K-means technique to select cluster heads.

Early versions of the LEACH protocol relied on probabilistic cluster-head selection methods. Selecting an inappropriate node to serve as a cluster head might rapidly drain its energy. Newer variants of LEACH address this issue by integrating local metrics, such as distance or residual energy, into the selection process. However, these newer variants are susceptible to premature convergence to local optima.

Chain-based and tree-based routing protocols are data routing paradigms that focus on constructing optimal routes from distant nodes to the sink rather than specific clusters. To improve the LEACH transmission protocol, Lindsey et al. [20] introduced the Power-Efficient Gathering in Sensor Information Systems (PEGASIS) technique. The key objective of PEGASIS is to reduce the energy spent on communication by forming chain-based routes instead of clusters. To tackle the issues of inefficient leader node selection and non-uniform chain formation in PEGASIS, Abose et al. [21] introduced the Improved Energy-Efficient Anytime Optimistic (IEEAO) protocol in which the leader is dynamically selected based on its energy, while Wang et al. [22] employed Jain’s Fairness Index (JFI) as a decision metric.

Chain-based routing protocols rely on a single linear path for transmitting data from distant nodes to the gateway, which can lead to latency and a high dependency on that single link. To overcome this issue, tree-based routing methods are proposed. Unlike chain-based approaches, tree-based structures construct multiple branches connecting distant nodes to a gateway or the base station. The tree-like nature of these structures enhances fault tolerance and load balancing. To optimize the energy consumption and resolve the energy hole problem in WSN, Fan et al. proposed the energy-balanced path tree-based clustering and routing algorithm (EBPT-CRA) in [15]. The Energy-balanced path trees (EBPT) are constructed using Dijkstra’s algorithm, with the nodes’ residual energy and the energy required for data transmission between the sender and receiver as the path cost. To select the optimal cluster head, this study introduces the concept of node convergence betweenness (NCB), a metric that quantifies a node’s centrality in the network and the number of nodes in its subtree.

Similar to LEACH techniques, chain- and tree-based protocols use local metrics to construct the transmission path from the source node to the destination, making these techniques susceptible to premature convergence to local optima.

### 2.2. Bio-Inspired Clustering and Routing Methods

Bio-inspired algorithms have proven to be useful for optimizing energy consumption in wireless sensor networks. Genetic algorithm (GA), ant colony optimization (ACO), and particle swarm optimization (PSO) are among the most prominent algorithms adapted to address data routing in wireless sensor networks.

Kedi et al. [23] proposed a two-level genetic algorithm that resolves the problem of the cluster head selection and the routing path selection by selecting the best possible set of nodes that can serve as cluster heads. The second-level GA serves as a subroutine to determine the most energy-efficient route for data from the set of selected cluster heads to the base station. Despite its efficiency in clustering and data routing in WSN, the GA algorithm is still prone to getting trapped in local optima. To overcome this issue, Jayachandran et al. [24] proposed a hybrid approach that combines the genetic algorithm (GA) to select the cluster head and Harris hawk optimization (HHO) to find the most efficient routes between the CHs and the base station.

Wang et al. [25] introduced a novel pseudo-random proportional rule to optimize the state transition formula to improve the exploration of the ant colony optimization (ACO) algorithm. The inclusion of the residual node energy parameter ensures that path selection is based not only on path length but also on the node energy levels along the path. Similarly, Han et al. [26] introduced an optimized ACO to optimize the process of routing in WSNs for the Internet of Things (IoT) by adjusting pheromone concentration and transfer probability to account for nodes’ residual energy.

In [27], Harris et al. proposed a novel particle swarm optimization (PSO) algorithm to enhance energy efficiency in WSNs through optimized cluster head selection. The core idea in this approach is the use of a double-exponential adaptive inertia (DEAI), which balances global exploration and local exploitation, helping the algorithm escape the trap of local minima. Parvin et al. [28] proposed the use of a PSO-based clustering technique to solve the problem of residual nodes, the nodes that are left without joining any cluster. Initially, this approach selects cluster heads based on nodes’ residual energy and their centrality in the network. However, some nodes might still fail to join any cluster. This is where a new PSO iteration starts, with the priority given to these residual nodes to serve as cluster heads. Authors in [29] proposed a quantum particle swarm optimization fuzzy logic approach in which a quantum-based cluster head selection method is used with a fuzzy-logic based data routing path construction.

Bio-inspired routing protocols have significantly improved the cluster head selection and the inter-cluster routing, which has led to a significant energy optimization of WSN. However, these techniques often do not consider the intra-cluster as a primary clustering and routing metric, which may lead to over-utilizing intermediate relay nodes between a source node and its cluster head. Furthermore, the standard PSO-based clustering and routing techniques are prone to premature convergence.

### 2.3. Entropy and Similarity Metrics Clustering and Routing Methods

The concept of entropy, a measure of disorder or uncertainty in information theory, has been adopted to address various challenges in WSNs. Energy-balanced clustering and data routing in wireless sensor networks are among the challenges addressed by entropy. By measuring the entropy difference between two or more network states, intelligent decisions can be made to optimize energy consumption in WSNs. General entropy methods and Kullback–Leibler (KL) divergence methods are efficient for decision-making and detecting changes in network states. In contrast, similarity metrics, such as the adjusted Rand index, are primarily used as external validity measures.

Proceeding from the ability of general entropy to quantify the uncertainty in the data, Sahoo et al. [30] presented an entropy-weighted method integrated with a multi-criterion decision-making technique for cluster head selection. Manoharan et al. [31] used the relative entropy method to build the entropy-based bald eagle search (EBES) algorithm. In this method, the entropy value is used to select the most suitable node to relay the data to the next hop.

Kullback–Leibler (KL) divergence is a powerful mathematical tool to quantify the differences between two probability distributions. The ability to measure differences between probability distributions makes the KL divergence a perfect tool for comparing several states of the system. Detecting data anomalies between transmitted data through the drastic changes in the KL values between consecutive rounds of transmissions is used to reduce the cost of redundant transmission [32,33].

Similarity metrics are external metrics employed to quantify the similarity between two solutions: the achieved solution and a reference solution. In the context of WSN, similarity metrics are used to compare the cluster configuration obtained by a clustering algorithm with a reference or desired solution. Kayalaap et al. [13] employed the ARI metric to validate the clustering performance of four evolutionary algorithms: the genetic algorithm (GA), PSO, gray wolf optimization (GWO), and biogeography-based optimization (BBO) against a reference solution: the k-means algorithm. Similarly, Kanaujia et al. [14] used the ARI metric as an external validation metric to assess the accuracy of their proposed clustering method, robust glowworm swarm clustering (RGSC), against the ground-truth solution, i.e., a known true cluster assignment.

Entropy and similarity-based methods have been mainly used either to measure the network’s spatial variance or as a passive diagnostic tool to detect the level of similarity between two clustering states. As seen from the literature review, we can conclude that traditional clustering and routing techniques, such as LEACH and tree-based clustering techniques, are prone to premature convergence to local minima. The metaheuristic clustering and routing techniques are often susceptible to premature convergence. Although some recent studies employed statistical metrics to measure the network variance, these tools are merely used as passive diagnostic tools. This study employs sophisticated tree-based clustering and routing as a baseline for a swarm intelligence to achieve global optimization of the network. Crucially, we shifted the use of ARI from a simple diagnostic tool to an early-stage detection mechanism to avoid premature convergence and guide the swarm to new search areas.

## 3. Materials and Methods

The proposed approach, ARI-DPSO, combines swarm intelligence with an energy-aware clustering and routing model to maximize the lifetime of wireless sensor networks. A fundamental concept in maximizing the lifetime of WSNs is the establishment of energy-efficient clustering and routing structures. To achieve this goal, the ARI-DPSO proposes a minimum-energy routing model to optimize the energy consumption of each node in each operational round. The discrete PSO is employed to find the optimal clustering configuration, using an ARI-derived dissimilarity mechanism to introduce a random perturbation component $c_{3}$ to prevent premature convergence.

### 3.1. Radio Model

For the purpose of evaluating our proposed routing protocol, we assume that energy depletion follows the first-order radio model as in [15]. In this model, an amount of energy $E_{\mathrm{tx}}$ required to send *l*-bit of data over a transmission distance *d* depends on whether *d* exceeds a threshold distance $d_{0}$:

(1) $$E_{\mathrm{tx}}\left(l,d\right)=\left\{\begin{array}{cc} l\;\cdot \;E_{\mathrm{elec}}+l\;\cdot \;\epsilon_{\mathrm{fs}}\;\cdot \;d^{2}, & \;\mathrm{if}\;d<d_{0}\\ l\;\cdot \;E_{\mathrm{elec}}+l\;\cdot \;\epsilon_{\mathrm{amp}}\;\cdot \;d^{4}, & \;\mathrm{if}\;d\geq d_{0} \end{array}\right..$$

The constant $\epsilon_{\mathrm{fs}}$ represents the amount of energy needed by the transmit amplifier under the free space propagation model, while $\epsilon_{\mathrm{amp}}$ represents the energy needed by the transmit amplifier under the multi-path propagation model. The threshold distance $d_{0}=\sqrt{\frac{\epsilon_{\mathrm{fs}}}{\epsilon_{\mathrm{amp}}}}$, and the energy consumed by the sensor hardware for data processing is $E_{\mathrm{elec}}$. The amount of energy required to receive *l*-bits of data is

(2) $$E_{\mathrm{rx}}=l\;\cdot \;E_{\mathrm{elec}}.$$

As explained in Equation (1), the amount of energy required to transmit over a distance *d* beyond the threshold $d_{0}$ escalates with the fourth power of the distance $d^{4}$. To avoid transmitting over long distances, we restricted the transmission range $d_{\mathrm{tx}}$ to be less than the threshold $d_{0}$. Thus, this restriction enables the data load to be distributed across energy-efficient paths.

### 3.2. Clustering and Optimal-Energy Routing

In this section, we present the network hierarchy and the method used to construct multi-hop, energy-optimal paths of the network. The main objective is to minimize the energy cost of the routing paths, as this minimized cost is used to evaluate the fitness of the proposed discrete PSO, as formalized in Algorithm 1. Table 1 explains the terms used in Section 3.2.

**Table 1:** Symbols used in the clustering and routing procedure.

Symbol	Meaning
$d_{\mathrm{tx}}$	One hop transmission range (m)
*l*	Packet size (bits)
$E_{\mathrm{init}}$	Initial energy per node (J)
$E_{\mathrm{elec}}$	Radio electronics energy (J/bit)
V	set of all sensor nodes
G	set of all gateway nodes
$C_{k}$	cluster
parent(i)	Next-hop node for node *i* on its path to the sink S
f(i)	data flow (packets) handled by node *i*
E(i)	Total energy consumed by node *i* per round
L(i)	Lifetime of node *i* (rounds)
$L_{\min}$	Minimum lifetime of any node in a cluster


**Algorithm 1** Minimum-energy path construction.**Require:** Node positions, Sink *S*, parameters $\left(d_{\mathrm{tx}},l,E_{\mathrm{init}},E_{\mathrm{elec}},\epsilon_{\mathrm{fs}},d_{0}\right)$
**Ensure:** Parent mapping parent( · ), Node lifetimes L( · ), Min. Lifetime $L_{\min}$

  1:

$G\leftarrow \{u|d\left(u,S\right)\leq d_{\mathrm{tx}}\}$



▹ Identify all nodes that can reach the sink: Gateways

  2:Partition nodes (V) into $d_{\mathrm{tx}}$-connected clusters $\left\{C_{k}\right\}$

  3:Initialize parent(i)←⊥ for all i∈V

  4:**for** each component $C_{k}$ with $C_{k}\cap G\neq \emptyset$ **do**

  5:   Order nodes $i\in C_{k}$ by decreasing d(i,S)

▹ Process farthest nodes first to prevent loops

  6:   **for** each $i\in C_{k}$ **do**

  7:    **if** i∈G **then**

  8:     parent(i)←S; continue 

▹ Gateway route directly to sink

  9:    **end if**
▹ Minimum-Energy Path Selection (Dijkstra)

10:    $\left({\mathrm{cost}}_{\mathrm{best}},\pi_{\mathrm{best}},g^{⋆}\right)\leftarrow \left(+\infty ,⊥,⊥\right)$

11:    **for** each gateway $g\in C_{k}\cap G$ **do**

12:     π←Dijkstra(i→g) over edges (x,y) with $d\left(x,y\right)\leq d_{\mathrm{tx}}$
▹ Dijkstra uses link_cost as edge weight (energy for 1 packet)

   $\mathrm{link\_cost}\left(x,y\right)=\left(E_{\mathrm{elec}}\;\cdot \;l+l\;\cdot \;\epsilon_{\mathrm{fs}}{\left(d\left(x,y\right)\right)}^{2}\right)+\left(E_{\mathrm{elec}}\;\cdot \;l\right)$

13:     **if** π=⊥ **then** continue

14:     **end if**

15:     ${\mathrm{cost}}_{\mathrm{path}}\leftarrow \sum_{(x,y)\in \pi }\mathrm{link\_cost}\left(x,y\right)$

▹ Cumulative energy cost of the multi-hop path

16:     ${\mathrm{cost}}_{\mathrm{total}}\leftarrow {\mathrm{cost}}_{\mathrm{path}}$

17:     **if** ${\mathrm{cost}}_{\mathrm{total}}<{\mathrm{cost}}_{\mathrm{best}}$ **then**

18:       $\left({\mathrm{cost}}_{\mathrm{best}},\pi_{\mathrm{best}},g^{⋆}\right)\leftarrow \left({\mathrm{cost}}_{\mathrm{total}},\pi ,g\right)$

19:     **end if**

20:    **end for**

21:    **if** $g^{⋆}\neq ⊥$ **then**

22:     Set parent(i) to first hop in $\pi_{\mathrm{best}}$

▹ Parent $p_{i}$ is the first hop of the optimal path

23:▹ The rest of the path is determined when intermediate nodes are processed: the sequential order

24:    **end if**

25:   **end for**

26:
**end for**


▹ Data Flow Aggregation and Lifetime Calculation

27:Initialize flow f(i)←1 for all i∈V

28:Define children(i)={c∣parent(c)=i}

29:Push flow bottom-up: $f\left(p\right)\leftarrow f\left(p\right)+\sum_{c\in \mathrm{children}(p)}f\left(c\right)$

30:**for** each node *i* with parent(i)≠⊥ **do**

31:   $E_{\mathrm{rx}}\leftarrow E_{\mathrm{elec}}\;\cdot \;l\;\cdot \;\sum_{c\in \mathrm{children}(i)}f\left(c\right)$

▹ Reception energy based on aggregated flow

32:   $p_{i}\leftarrow \mathrm{parent}\left(i\right)$

33:   $d_{i}\leftarrow d\left(i,p_{i}\right)$ (or d(i,S) if $p_{i}=S$)

34:   $E_{\mathrm{tx}}\leftarrow \left(E_{\mathrm{elec}}\;\cdot \;l+l\;\cdot \;\epsilon_{\mathrm{fs}}{\left(d_{i}\right)}^{2}\right)\;\cdot \;f\left(i\right)$

35:   $E\left(i\right)\leftarrow E_{\mathrm{rx}}+E_{\mathrm{tx}}$

▹ Total Energy Burden

36:   $L\left(i\right)\leftarrow E_{\mathrm{init}}/E\left(i\right)$

▹ Node Lifetime

37:
**end for**


38:

$L_{\min}\leftarrow \min_{i\in V\wedge \mathrm{parent}(i)\neq ⊥}L\left(i\right)$



▹ Network Fitness (FND lifetime)

39:**return** $\mathrm{parent}\left(\;\cdot \;\right),L\left(\;\cdot \;\right),L_{\min}$



#### 3.2.1. Network Structure and Cluster Formation

The network structure categorizes nodes into two layers based on their connectivity and ability to transmit data to the sink S. The members of the set of gateway nodes G are the only nodes that can directly transmit to the sink. A crucial component of this structure is the concept of a gateway node. A gateway node is any node *u* within the transmission range $d_{\mathrm{tx}}$ of the sink S. The clustering routine partitions the entire set of nodes V in the network into several disjoint clusters $C_{k}$ with the number of clusters strictly less than the number of gateway nodes. Restricting the number of clusters to be less than the number of gateway nodes guarantees that there is a sufficient number of gateway nodes to equip each cluster with at least one gateway node. The clustering procedure is described as follows:

(3) $$\begin{array}{c} V=\underset{k}{⋃}C_{k}\;\mathrm{such}\;\mathrm{that}\;C_{i}\cap C_{j}=\emptyset \;\mathrm{for}\;i\neq j\\ \mathrm{and}\;\forall x,y\in C_{k},x\;\mathrm{is}\;\mathrm{connected}\;\mathrm{to}\;y. \end{array}$$

The term $C_{k}$ denotes the set of nodes forming a cluster; $V={⋃}_{k}C_{k}$ denotes that each node in the set $C_{k}$ is a member of a cluster; $C_{i}\cap C_{j}=\emptyset \;\mathrm{for}\;i\neq j$ dictates that no node can belong to two distinct clusters, ensuring that the clusters form a partition of the set of nodes V. The term $\forall x,y\in C_{k},x\;\mathrm{is}\;\mathrm{connected}\;\mathrm{to}\;y$ states that any two nodes *x* and *y* in a cluster $C_{k}$ are connected either directly if they are within the transmission range or via a multi-hop path connection. In addition to the connectivity constraints in Equation (3), a cluster is considered valid only if it contains at least a gateway node:

(4) $$C_{k}\cap G\neq \emptyset .$$

To ensure that only valid clusters are formed, each formed cluster is checked for the connectivity of its members and for the presence of at least a gateway node. Cluster configurations that fail this check are reset, and the cluster generation mechanism will retry the generation process until a valid cluster configuration is achieved.

The data is transmitted from distant nodes in the cluster to the sink via the gateway in multi-hop paths. To prevent routing loops in the data paths, this approach sorts all non-gateway nodes in a cluster by their decreasing distance to the sink. Establishing the network structure requires defining the parent–child relationship between all nodes in a routing path, where the children(i) is the set of nodes that set the node *i* as their parent:

(5) children(i)={c∣parent(c)=i}.

Unlike the gateway nodes, which have the sink as their parent, the parent–child relationship between non-gateway nodes is dynamic and depends on the minimum-energy path selection, which we invoke in the next subsection.

#### 3.2.2. Minimum-Energy Path Selection

Before presenting the details of the minimum-energy path selection, it is relevant to highlight that the energy consumption in this section is calculated using the first-order radio model explained in Section 3.1.

An energy-optimal multi-hop path is a path that connects every non-gateway node *i* in a cluster $C_{k}$ to a gateway with the minimum energy cost. To achieve this goal, we treat every cluster as a graph, $G_{k}=\left(C_{k},E_{k}\right)$ where $E_{k}$ is the set of adjacent edges between two nodes (x,y) (i.e., $\forall x,y\in C_{k}:d\left(x,y\right)\leq d_{\mathrm{tx}}$). Then, Dijkstra’s algorithm is employed to select the path that minimizes the energy cost of transmitting from node *i* to every candidate *g* within the cluster. The total energy dissipated for the transmission and reception of a data packet of length *l* represents the link cost:

(6) $$\mathrm{link\_cost}\left(x,y\right)=\left(E_{\mathrm{elec}}\;\cdot \;l+\epsilon_{\mathrm{fs}}\;\cdot \;l\;\cdot \;d{\left(x,y\right)}^{2}\right)+\left(E_{\mathrm{elec}}\;\cdot \;l\right).$$

We evaluate all the possible paths Π(i→g) linking a node *i* to a gateway *g* and select the least costly one. The cost of a path π∈Π(i→g) is the sum of its link costs:

(7) $${\mathrm{cost}}_{\mathrm{path}}\left(\pi \right)=\sum_{(x,y)\in \pi }\mathrm{link\_cost}\left(x,y\right).$$

A node *i* must select the gateway that can be reached with the minimum path cost $g^{⋆}$ from several possible candidate gateways in each cluster:

(8) $$g^{⋆}=\underset{g\in C_{k}\cap G}{\arg\min}\left(\min_{\pi \in \Pi (i\to g)}{\mathrm{cost}}_{\mathrm{path}}\left(\pi \right)\right).$$

The path that links the node *i* to the optimal gateway is the best path $\pi_{\mathrm{best}}$:

(9) $$\pi_{\mathrm{best}}=\underset{\pi \in \Pi (i\to g^{⋆})}{\arg\min}\left({\mathrm{cost}}_{\mathrm{path}}\left(\pi \right)\right).$$

The first hop in the path is set to be the parent of node *i*.

Data transmission from a distant node in a cluster $C_{k}$ to the sink follows a parent-to-parent sequence. The complete path from a node $i\in C_{k}$ to the sink is formed by a sequence of intermediate nodes $P_{i}=\langle n_{0},n_{1},\ldots ,n_{m},S\rangle$. This sequence begins at the source node *i*, denoted as $n_{0}$ in the sequence above, and it selects its immediate parent $p_{i}$ as the first relay node in the best path $\pi_{\mathrm{best}}$. The node $p_{i}$ is the source node in this stage of the sequential process; it computes its path cost, selects its optimal gateway, and determines its immediate parent. Following this chain of transmission, the *k*-th node in the chain is always the optimal parent of the preceding node: $n_{k}=p_{n_{k-1}}$. This sequence of parent-to-parent transmission continues until the optimal gateway node ($n_{m}=g^{⋆}$), which takes the sink as its parent $p_{n_{m}}=S$. Since the valid clusters are formed with connected node members as in Equation (3), and each cluster is associated with at least a gateway as per Equation (4), at the end of the sequential order process of parent selection, all the non-gateway nodes would have established a path to the sink.

**Figure 1:**
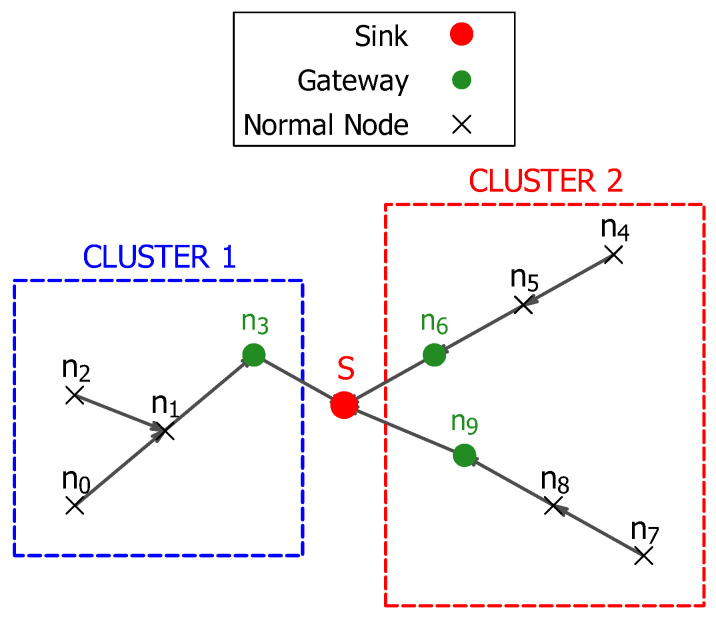
Cluster layout and intra-cluster data routing.

The sequential parent-to-parent data transmission guarantees that even the most distant nodes in each cluster can find a multi-path, energy-efficient route to their corresponding gateway. Figure 1 shows the cluster layout and the intra-cluster data routing from distant nodes to the gateways inside each cluster.

#### 3.2.3. Data Flow Aggregation and Lifetime Calculation

At this stage, the actual energy expenditure of a node *i* is computed based on its parent–child routing path structure. Then, this energy calculation is used to compute the overall network lifetime.

The total data flow of a node *i* depends on the role it plays in the routing path structure. The data flow f(i) of each node is initialized to the value of one packet of data since each node must transmit to its next destination. Parent nodes *p* have to account for the energy burden of receiving from their children and transmitting the sum of their initial flow f(i) and their children’s flows f(c). The total amount of energy, E(i), consumed by each node *i* in each round is computed using the cumulative data and by employing the first-order radio model, as follows:

(10) $$E\left(i\right)=E_{\mathrm{rx}}+E_{\mathrm{tx}}.$$

The term $E_{\mathrm{rx}}$ is the energy consumed by a node *i* to receive data from all its children:

(11) $$E_{\mathrm{rx}}=E_{\mathrm{elec}}\;\cdot \;l\;\cdot \;\sum_{c\in \mathrm{children}(i)}f\left(c\right).$$

The notion of children(i) is defined by Equation (5). The second term of the per-round energy consumption E(i), the energy required to transmit the accumulated flow f(i) of the node *i* to its parent $p_{i}$:

(12) $$E_{\mathrm{tx}}=\left(E_{\mathrm{elec}}\;\cdot \;l+\epsilon_{\mathrm{fs}}\;\cdot \;l\;\cdot \;d{\left(i,p_{i}\right)}^{2}\right)\;\cdot \;f\left(i\right).$$

The term $d(i,p_{i})$ denotes the distance between a node *i* and its parent $p_{i}$. The lifetime of each node L(i) is a function of its initial energy $E_{\mathrm{init}}$ and its energy consumed per round E(i) is

(13) $$L\left(i\right)=\frac{E_{\mathrm{init}}}{E(i)}.$$

The network lifetime is defined by the first node’s death:

(14) $$L_{\min}=\min_{i\in C_{k}}\left(\frac{E_{\mathrm{init}}}{E(i)}\right).$$

#### 3.2.4. Integrated Connectivity and Route Selection

The complete method of constructing connected data transmission routes is presented in Algorithm 1. Table 1 explains the terms used in Algorithm 1. The network hierarchy and cluster formation are established in (lines:1–9) in Algorithm 1, where gateway nodes G are determined based on their distance to the sink in (line:1). Then, the partition of the set of all nodes in the network V into a set of clusters in (line:2). The gateway nodes are assigned the sink as their immediate parent in (line:8), while non-gateway nodes are sorted in decreasing order by their distance from the sink to avoid routing loops in (line:5).

The minimum energy path selection is highlighted by the lines (lines:10–26), where a loop is launched in (line:11) to iterate through all gateway nodes G in a cluster $g\in C_{k}\cap G$, and the Dijkstra’s algorithm is applied to find the optimal path π of a node *i* over a single hop, in (line:12). The total path cost ${\mathrm{cost}}_{\mathrm{path}}$ is calculated as the total sum of all link costs of each hop in the path π in (line:15). The optimal gateway $g^{⋆}$ and the path that costs the minimum energy are selected in (lines:17–18), and the parent $p_{i}$ of the source node *i* is the first hop in π as in (line:22).

The final stage in this algorithm is the data flow aggregation and the lifetime calculation part covered in (lines:27–37). This stage begins by initializing the data flow f(i) of each node with a unit flow in (line:27), then an aggregation is performed to determine the cumulative flow handled by each node in (line:29). The total energy burden of each node E(i) is calculated in (line:35) as a sum of the energy needed to receive from *i*’s children (line:31) and the energy needed to transmit to its next parent (line:34). Finally, the network lifetime $L_{\min}$ is returned as the primary optimization factor in (line:39).

### 3.3. Optimization with Discrete-PSO

Initially, the clusters established in Section 3.2 are technically sufficient to guarantee data routing in the network. As the multi-hop paths in these clusters are constructed using methods that operate on local heuristics, they may produce a locally acceptable performance, but they may not achieve the true global optimum.

To efficiently cover a large search area, DPSO creates a large number of candidate solutions (particles), where each particle represents a complete solution, i.e., a complete set of cluster formations. At each iteration, a set of clusters is generated, and the paths are constructed in the same manner as in Section 3.2.1. To balance exploration and exploitation, the positions and velocities of the particles are updated based on their own best local and historical solutions.

#### DPSO vs. PSO

One of the main differences between conventional PSO methods and their discrete counterparts lies in the mechanisms for particle movement and velocity updates. Conventional PSO methods are designed to operate in a continuous search space, where a vector addition of real values governs their position and velocity updates. Conversely, the problem of partitioning nodes into clusters is discrete, where the particle’s position is defined by a vector that encodes the node’s membership in a cluster. The position vector, velocity vector, velocity update, and position update in the WSN clustering using DPSO are set as follows:

- Position Vector $X^{\left(p\right)}$: is the vector that stores the position of a particle *p* at each iteration *t*, by assigning each node *j* to its current cluster. The position vector of *N* sensor nodes is defined as: $X^{\left(p\right)}=\left\{x_{1}^{\left(p\right)},x_{2}^{\left(p\right)},\ldots ,x_{N}^{\left(p\right)}\right\}$, where $X^{\left(p\right)}\in {\left(Z^{+}\right)}^{N}$. In this context, the term $x_{j}^{\left(p\right)}\in Z^{+}$ is the ID of the cluster to which node *j* belongs in particle *p*.
- Velocity Vector (V): unlike the continuous vector in the PSO, the velocity vector in the DPSO stores a set of “Move” operators $V_{\mathrm{set}}$ that govern a node’s changes in membership from one cluster to another. A move *v* in the set of moves $V_{\mathrm{set}}$ is defined by the triplet:
  $$v=\langle j,c_{\mathrm{from}},c_{\mathrm{to}}\rangle ,$$
  where $c_{\mathrm{from}}$ is the current cluster ID of node *j*, derived from the position vector as: $c_{\mathrm{from}}=x_{j}^{\left(p\right)}$, and $c_{\mathrm{to}}$ is the target cluster ID for node *j*.
- Velocity update: the velocity vector $V_{p}^{t+1}$ is the aggregation of the influence of the inertia ω, the cognitive influence $c_{1}$ and the social influence $C_{2}$ via the set operator ⊕:
  (15) $$V_{p}^{t+1}=\omega ⊗V_{p}^{t}⊕c_{1}⊗\left(P_{\mathrm{best}}⊖X^{\left(p\right)}\right)⊕c_{2}⊗\left(g_{\mathrm{best}}⊖X^{\left(p\right)}\right).$$
  The operator ⊖ denotes the set of moves required to move the $X^{\left(p\right)}$ to the personal best solution $P_{\mathrm{best}}$ or the global best solution $g_{\mathrm{best}}$. The stochastic operator ⊗ filters the set of moves based on the values of the DPSO parameters: ω, $c_{1}$, and $c_{2}$. For instance, to approve the set of moves required by $c_{2}$ to move $X^{\left(p\right)}$ to $g_{\mathrm{best}}$, the stochastic operator ⊗ compares the value of $c_{2}$ to a random number rand(0,1) between 0 and 1. Each time a move in this set of moves is filtered, a new random number is generated, and a move is accepted if and only if the random number is less than $c_{2}$. This filtering process is applied to the set of moves required by $c_{1}$ to pull $X^{\left(p\right)}$ toward $P_{\mathrm{best}}$, and applied to the influence of the inertia ω. This filtering is formulated as follows:
  (16) $$c⊗V_{\mathrm{set}}=\left\{v|v\in V_{\mathrm{set}}\wedge \mathrm{rand}\left(0,1\right)<c\right\},\;$$
  where the term *c* denotes the influence of the DPSO terms: ω, $c_{1}$ and $c_{2}$. The operator ⊕ receives the set of all accepted moves and stores them in a sequence of node-cluster membership changes defining the update tendency of the current iteration. Note that, in this section, we detailed the velocity updates based on ω, $c_{1}$, and $c_{2}$ only; the introduction of the adaptive parameter $c_{3}$ will be detailed in Section 3.6.
- position update: we update the particle position by applying the list of stochastic moves in the velocity vector to the current position vector:
  (17) $$X_{t+1}^{\left(p\right)}=X_{t}^{\left(p\right)}⊗V_{p}^{t+1}.\;$$

### 3.4. Fitness Function

As stated in Section 1, this study aims to extend the longevity of the network by delaying the occurrence of the first node death. The lifetime of the network is determined by the first node death and is calculated by the definition (14). To achieve this goal, we formulate the fitness function of the ARI-DPSO to obtain the maximum lifetime of the network as follows:

(18) $$\mathrm{Fitness}=\max\left(0.0,\min_{i\in C_{k}}\left(\frac{E_{\mathrm{init}}}{E(i)}\right)\right).$$

The global and intelligent search capability of DPSO enables it to achieve better solutions than deterministic routes can achieve. However, the standard DPSO is associated with stagnation and getting stuck in the local minima as drawbacks.

### 3.5. The Adaptive Mechanism

The inertia ω, the personal best solution $P_{\mathrm{best}}$, and the global best solution $g_{\mathrm{best}}$ try to guide the swarm to a balanced exploration of new search areas as well as exploiting local search areas. However, in DPSO, the swarm is prone to stagnating in limited search areas as its particles tend to prematurely converge to the $g_{\mathrm{best}}$ found by the swarm so far. The $g_{\mathrm{best}}$ might not be the best global solution, and prematurely converging to this solution might have greater negative effects on problems that require exhaustive search to find optimal solutions. From a computational complexity perspective, the number of ways to organize a set of nodes into a set of paths is similar to the number of ways to partition a set of nodes into a set of non-empty clusters. This process is described by the Stirling number of the second kind as in [34], which yields a very large number of possible path configurations.

To alleviate the complication of premature convergence, this study introduces a proactive and adaptive perturbation coefficient $c_{3}$ to the parameters of the DPSO. The parameter $c_{3}$ injects a source of controlled randomness into a particle based on its similarity with the global best solution $g_{\mathrm{best}}$. At each iteration, each generated particle is compared to the global best solution to calculate the coefficient of perturbation $c_{3}$ required to adequately repulse the current particle from the $g_{\mathrm{best}}$. To avoid stagnation, a current particle with a high level of similarity to the $g_{\mathrm{best}}$ requires a high level of perturbation, and hence a higher (but controlled) $c_{3}$. Conversely, a current particle that exhibits a minimum level of similarity to the $g_{\mathrm{best}}$ needs minimum perturbation. The similarity between current particles and the $g_{\mathrm{best}}$ is measured using the ARI metric, and the level of this similarity dictates the required perturbation influence of perturbation element $c_{3}$.

#### 3.5.1. The ARI-Guided Perturbation

To present the ARI metric, we must first introduce an essential concept in ARI computation: the contingency matrix. The contingency matrix counts the number of nodes in agreement between two clustering configurations, the current particle $X^{\left(p\right)}$ and the global best solution $g_{\mathrm{best}}$. Table 2 presents an assignment of six nodes: node (*c*) to node (*h*) to their respective clusters: Cluster *X*, Cluster *Y*, and Cluster *Z* in $X^{\left(p\right)}$ and their assignment to the clusters, *P*, *Q* and *R* in $g_{\mathrm{best}}$.

The contingency table is presented in Table 3, where the number of node agreements between both configurations is counted. To count the number of nodes’ agreement in both clustering configurations, we start with the members of cluster *X* in $X^{\left(p\right)}$: node *c* and node *d*, then we count their presence in the clusters *P*, *Q* and *R* in $g_{\mathrm{best}}$. Since the node *c* is present in the cluster *X* in $X^{\left(p\right)}$ and in Cluster *P* in $g_{\mathrm{best}}$, we mark one intersection between Cluster *X* and Cluster *P* in the entry cell $n_{X,P}$ as shown in the cell entry in Table 3, $n_{X,P}=1$, where one is the number of agreements between both clusters. Similarly, the node *d* is present in the cluster *Q*, which yields an entry cell $n_{X,Q}=1$ and entry cell $n_{X,R}=0$ with no count of agreement. Note that the nodes *e* and *f* are present in the cluster *Y* and the cluster *Q*, which makes both clusters have an agreement of two in the entry cell $n_{Y,Q}=2$. The procedure is repeated for the remaining nodes in the rest of the rows and columns in the contingency table. These entry cells that count the number of agreements between clustering configurations are referred to as the intersection count $n_{i,j}$. The row sum and the column sum $a_{i}$ and $b_{j}$ are the total sizes of the *i*-th and *j*-th clusters in $X^{\left(p\right)}$ and $g_{\mathrm{best}}$, respectively.

Following the derivation by Hubert et al. [12], and with the help of Table 3, we present the formal definition of the ARI metric in the Equations (19)–(22):

(19) $$\mathrm{ARI}=\frac{\mathrm{Index}-\mathrm{Expected}\;\mathrm{Index}}{\mathrm{Max}\;\mathrm{Index}-\mathrm{Expected}\;\mathrm{Index}},$$

where

(20) Index=∑i,jnij2,

is the number of pairs of elements common to cluster *i* in $X^{\left(p\right)}$ and cluster *j* in $g_{\mathrm{best}}$,

(21) Max Index=12∑iai2+∑jbj2,

where $a_{i}$ and $b_{j}$ are the total sizes of the *i*-th and *j*-th clusters in $X^{\left(p\right)}$ and $g_{\mathrm{best}}$, respectively, and

(22) Expected Index=∑iai2∑jbj2N2,

where *N* is the total number of nodes.

The adaptive coefficient of perturbation $c_{3}$ is computed as a function of the ARI metric. Equation (19) reveals that the ARI can theoretically have negative values in the cases where Expected Index > Index. Table 4 shows that Expected Index is the number of node pairs expected to agree purely by random chance, and Index is the actual number of agreeing node pairs. This means that a negative value of ARI denotes that $X^{\left(p\right)}$ and $g_{\mathrm{best}}$ are extremely dissimilar to the extent that even a random guess would have resulted in more agreeing pairs of nodes in $X^{\left(p\right)}$ and $g_{\mathrm{best}}$. Therefore, Hubert et al. [12] stated that such negative ARI values have no practical use and offer no practical interpretations. In this study, allowing such non-useful ARI values leads to producing a negative perturbation coefficient, which could have a destructive effect on the algorithm logic. Therefore, we set the minimum value of the ARI score to zero, and we compute $c_{3}$ as:

(23) $$c_{3}=C_{\mathrm{cap}}\;\cdot \;\max\left(0,\mathrm{ARI}\right),$$

where $C_{\mathrm{cap}}$ is a hyperparameter.

Algorithm 2 presents a summary of the steps needed to compute the ARI, while Table 4 outlines the components used in the ARI calculation. The following example presents the detailed steps required to compute the perturbation coefficient using ARI.
**Algorithm 2** Calculation of the adaptive perturbation coefficient $c_{3}$.**Require:** Assignment Vector $X^{\left(p\right)}$, Global Best Vector $g_{\mathrm{best}}$, Total number of nodes *N*, Max Perturbation Cap $C_{\mathrm{cap}}$.
**Ensure:** Adaptive Perturbation Coefficient $c_{3}$
▹ Step 1: Compute the Contingency Matrix and Marginals
  1:Initialize the contingency matrix *M* where $M\left[i,j\right]\leftarrow n_{i,j}$  2:Calculate Marginal Sums:  3:$a_{i}\leftarrow \sum_{j}n_{ij}$
▹ Cluster size in $X^{\left(p\right)}$
  4:$b_{j}\leftarrow \sum_{i}n_{ij}$
▹ Cluster size in $g_{\mathrm{best}}$

▹ Step 2: Calculate Core Pair counts
  5:PN←N2
▹ Total number of pairs
  6:Pa←∑iai2
▹ Total pairs within $X^{\left(p\right)}$ clusters
  7:Pb←∑jbj2
▹ Total pairs within $g_{\mathrm{best}}$ clusters
  8:Index←∑i,jnij2
▹ Observed Agreement Index

▹ Step 3: Calculate Normalization Terms
  9:$\mathrm{Expected}\;\mathrm{Index}\leftarrow \frac{P_{a}\;\cdot \;P_{b}}{P_{N}}$
▹ Agreement expected by chance
10:$\mathrm{Max}\;\mathrm{Index}\leftarrow \frac{1}{2}\left(P_{a}+P_{b}\right)$
▹ Maximum Possible agreement

▹ Step 4: Calculate Adjusted Rand Index (ARI)
11:$\mathrm{ARI}\leftarrow \frac{\mathrm{Index}-\mathrm{Expected}\;\mathrm{Index}}{\mathrm{Max}\;\mathrm{Index}-\mathrm{Expected}\;\mathrm{Index}}$
▹ Step 5: Determine Adaptive Coefficient $c_{3}$
12:Similarity←max(0,ARI)
▹ Round to 0 to prevent negative perturbation
13:$c_{3}\leftarrow C_{\mathrm{cap}}\;\cdot \;\mathrm{Similarity}$14:**return** $c_{3}$

#### 3.5.2. Example

Proceeding from the clustering configuration shown in Table 2, the following example illustrates the steps involved in computing the ARI-based perturbation coefficient $c_{3}$. We will apply the 5 steps to compute the coefficient of perturbation $c_{3}$ as highlighted by Algorithm 2, with the definitions of the terms explained in Table 4.

Step 1: The contingency matrix is presented in Table 3.

Step 2: Core pair counts

- Total number of node pairs ($P_{N}$) for a network of total number of nodes N=6 is
  PN=N2=62=6×52=15.
- Row Pairs Sum ($P_{a}$): (Total pairs in $X^{\left(p\right)}$ clusters, with $a_{i}$ obtained from the Row Sum in Table 3)
  Pa=∑iai2=22+22+22=1+1+1=3.
- Column Pairs Sum ($P_{b}$): (Total pairs within $g_{\mathrm{best}}$ clusters, with $b_{j}$ obtained from the column sum in Table 3)
  Pb=∑jbj2=12+32+22=0+3+1=4.
- Index (Observed Agreement): Compute the pairs in the same cluster in both solutions by taking the sum of nij2 in all intersection cells in Table 3
  Index=∑i,jnij2 =12+12+02+02+22  +02+02+02+22 =0+0+0+0+1+0+0+0+1 =2.

Step 3: Calculate normalization terms

- Expected Index (Agreement by chance)
  $$\mathrm{Expected}\;\mathrm{Index}=\frac{P_{a}\;\cdot \;P_{b}}{P_{N}}=\frac{3\;\cdot \;4}{15}=\frac{12}{15}=0.8.$$
- Max Index (Maximum Possible Agreement):
  $$\mathrm{Max}\;\mathrm{Index}=\frac{1}{2}\left(P_{a}+P_{b}\right)=\frac{1}{2}\left(3+4\right)=\frac{7}{2}=3.5.$$

Step 4: Calculate adjusted Rand index (ARI)

$$\mathrm{ARI}=\frac{\mathrm{Index}-\mathrm{Expected}\;\mathrm{Index}}{\mathrm{Max}\;\mathrm{Index}-\mathrm{Expected}\;\mathrm{Index}}=\frac{2-0.8}{3.5-0.8}=\frac{1.2}{2.7}\approx 0.4444.$$

Step 5: Determine adaptive coefficient $c_{3}$ From Equation (23), the coefficient of perturbation is

$$c_{3}=C_{\mathrm{cap}}\;\cdot \;\max\left(0,\mathrm{ARI}\right).$$

### 3.6. Adaptive Perturbation Mechanism and Swarm Dynamics

The introduction of the perturbation component $c_{3}$ aims to enhance the exploration of the swarm and to avoid the trap of the premature convergence of the discrete particle swarm optimization search. This parameter is introduced to complement the influence exerted by ω, $c_{1}$, and $c_{2}$ on the moves required for the velocity updates $V_{p}^{t+1}$ of the discrete swarm:

(24) $$V_{p}^{t+1}=\left(\omega ⊗V_{p}^{t}\right)⊕\left(c_{1}⊗\left(P_{\mathrm{best}}⊖X^{\left(p\right)}\right)\right)⊕\left(c_{2}⊗\left(g_{\mathrm{best}}⊖X^{\left(p\right)}\right)\right)⊕\left(c_{3}⊗V_{\mathrm{random}}\right).$$

The aim of introducing the perturbation element is to drive the current particle $X^{\left(p\right)}$ to a randomly selected particle $X^{\left(k\right)}$. The element $V_{\mathrm{random}}$ contains all the set of moves required to drive $X^{\left(p\right)}$ toward $X^{\left(k\right)}$, meaning that for every node *j*, the move $\langle j,x_{j}^{\left(p\right)},x_{j}^{\left(k\right)}\rangle$ is contained in $V_{\mathrm{random}}$. However, not any triggered move for a node will be added to the velocity update vector. Similar to the filtering method in Equation (16), the ⊗ operator in $c_{3}⊗V_{\mathrm{random}}$ filters these moves by comparing the value of the triggering component $c_{3}$ to a random number between (0,1). A move $\langle j,x_{j}^{\left(p\right)},x_{j}^{\left(k\right)}\rangle$ to drive $X^{\left(p\right)}$ toward $X^{\left(k\right)}$ is added to the velocity update vector only if $\mathrm{rand}\left(0,1\right)<c_{3}$ and $x_{j}^{\left(p\right)}\neq x_{j}^{\left(k\right)}$. To combat premature convergence, any high level of similarity between $X^{\left(p\right)}$ and $g_{\mathrm{best}}$ is rescued by a high perturbation to drive the particle away from the premature convergence search area. A high level of similarity between $X^{\left(p\right)}$ and $g_{\mathrm{best}}$ is reflected through a high value of ARI, which can reach the value 1 in the cases of a perfect match. Since $c_{3}$ depends only on the values of ARI and a constant $C_{\mathrm{cap}}$ as shown in Equation (23), a high value of ARI yields a high value of $c_{3}$. As a result, the majority of the moves $\langle j,x_{j}^{\left(p\right)},x_{j}^{\left(k\right)}\rangle$ to drive $X^{\left(p\right)}$ toward $X^{\left(k\right)}$ triggered by $c_{3}$ will be added to the velocity update vector because a high value of $c_{3}$ is likely to pass the stochastic filter (i.e., $\mathrm{rand}\left(0,1\right)<c_{3}$).

To resolve the conflict of priority between the DPSO components when triggering a single node, we use a priority sequence triggering mechanism. That means if multiple components (ω, $c_{1}$, $c_{2}$, and $c_{3}$) all try to trigger a move of a node *j* in the same iteration, the priority is given to these components by their order of execution in the velocity update: the inertia, the social influence, the cognitive influence, and least prioritized is, the perturbation coefficient.

### 3.7. Connectivity Validation

The ARI-DPSO is highly dependent on the rules set by the clustering and routing mechanism in Section 3.2.1, as a cluster configuration will be deemed valid only if it meets the condition of connectivity and the ability to transmit to the sink as enforced by Equations (3) and (4). An aggressive perturbation induced by high values of $c_{3}$ could lead to situations where a large number of created particles violates the constraints of valid cluster configurations. Particles that violate these connectivity constraints are deemed to have a null lifetime and are discarded from the swarm. To avoid this situation, we introduced a tunable parameter $C_{\mathrm{cap}}$ to control the aggressiveness of the perturbation factor $c_{3}$ to a moderate level that still produces an adequate level of exploration without being destructive to the network stability.

### 3.8. Complexity Analysis

To determine the total complexity of the ARI-DPSO, we need to examine the complexity of the main components involved in a single iteration and then identify the dominant complexity term. Let the number of nodes *N* be the cardinality of the set of all sensor nodes N=|V|. The complexity analysis of the major components of the ARI-DPSO algorithm is presented as follows:

- Cluster formation: the cluster formation requires the particle assignment vector to run a single traversal over all nodes in O(N).
- Cluster connectivity: A cluster is deemed valid only if the reachability between its member nodes is guaranteed. A breadth-first search (BFS) checks the reachability between all consecutive node pairs in a cluster, within $O(n_{c}^{2})$, where $n_{c}$ is the number of nodes in a cluster. The extreme case occurs when only a single cluster is formed, and hence all nodes are contained in this single cluster, yielding a complexity of $O(N^{2})$.
- Multi-hop path selection: As established in Section 3.2.2, the non-gateway nodes employ Dijkstra’s algorithm to select the most efficient path to the sink. In the extreme case where all nodes form a single cluster, that cluster contains O(N) nodes. The Dijkstra algorithm is executed for every member node on the O(N) vertices, and it is applied on a graph constrained by the connectivity check that requires $O(N^{2})$ in the extreme case. Thus, the total complexity required for this component is $O\left(N\right)\times O\left(N^{2}\right)=O\left(N^{3}\right)$.
- Data Flow Aggregation: In this component, each node sends aggregated data from its children and transmits its sensed data and the aggregated data to the next destination, which is a linear operation and requires O(N) time.
- Lifetime Calculation: this component requires iteration through each member node to determine its transmission and reception energy in O(N).
- Clustering and data routing: the complexity of these major components in the clustering and data routing phase is combined as: $O\left(N\right)+O\left(N^{2}\right)+O\left(N^{3}\right)+O\left(N\log N\right)+O\left(N\right)$. The cubic term $O(N^{3})$ is the dominant term in the clustering and data routing phase.
- The overall ARI-DPSO complexity: at each iteration, all particles *P* are formed and their lifetime is evaluated, which requires the cluster formation and the fitness evaluation. Thus, the complexity for each iteration is $O(P\;\cdot \;N^{3})$, and this per-iteration complexity is repeated over the number of iterations *T*. Thus, the entire complexity of the overall ARI-DPSO is: $O(T\;\cdot \;P\;\cdot \;N^{3})$. Although the velocity and position are updated for each iteration, their updates are executed in O(N), which is well dominated by the cubic term $O(N^{3})$ incurred by the cluster formation and the path finding.
- The complexity analysis of ω, $c_{1}$, and $c_{2}$: although the velocity and position are updated for each iteration, their updates are executed in O(N), which is well dominated by the cubic term $O(N^{3})$ incurred by the cluster formation and the path finding. The velocity updates comprise the inertia components ω, the cognitive influence component $c_{1}$, the social influence component $c_{2}$, and the perturbation element $c_{3}$. Computing the inertia components in the velocity updates simply requires iterating over the existing velocity vector to decide which set of moves to be reused in the next iteration, which is executed in O(N). The $c_{1}$ component computation requires comparing the current particle with the best personal solution, which requires a complexity of O(N), then generating the required set of moves to pull the current particle to the best personal solution executed at O(N). Thus, the total complexity of this component is still within O(N). The complexity of the $c_{2}$ influence follows the same method, with the distinction that the current particle is compared and pulled towards the global best solution, yielding the same complexity of O(N).
- The complexity analysis of the perturbation: the main difference between the complexity analysis of $c_{1}$, $c_{2}$ on one hand and the complexity analysis of $c_{3}$ on the other hand is that $c_{3}$ employs the ARI metric to compute the similarity between a current particle and the global best. The contingency table plays an important role in the ARI computation. The contingency table construction requires a single traversal of the node membership to compare their agreement in the $X^{\left(p\right)}$ and $g_{\mathrm{best}}$ and increment the intersection count $n_{i,j}$, the row sum and the column sum $a_{i}$ and $b_{j}$ as described in Section 3.5. This traversal over all nodes *N* is executed in a complexity of O(N) and constitutes the dominant term in the $c_{3}$ components computations. The remaining steps in the calculation of $c_{3}$ are either constant or cluster-count-based operations. Since each particle is compared to the $g_{\mathrm{best}}$ in each iteration, the computational cost of applying $c_{3}$ is O(P · N), which is still dominated by $O(N^{3})$ incurred by the cluster formation and the path finding.

With a complexity of $O(N^{3})$, the clustering and path-finding procedure is the most complexity-intensive component in the ARI-DPSO. This procedure is executed for every particle *P* for all iterations *T*; therefore, the complexity of the ARI-DPSO is simplified to $O(T\;\cdot \;P\;\cdot \;N^{3})$. The only difference between the ARI-DPSO and the standard DPSO is the introduction of the perturbation term $c_{3}$. However, the computation of $c_{3}$ and its influence on the velocity and position updates has a negligible effect on the complexity of the ARI-DPSO.

As seen from the complexity analysis, the computational complexity of ARI-DPSO is in the range of $O(N^{3})$, which makes it not feasible to be executed for real-time simulation for extremely dense networks. However, since this study mainly serves as a proof-of-concept of using ARI-DPSO to extend the network lifetime, and it is not a ready-to-deploy approach, we propose the following mechanisms to address the complexity issue:

- Centralized software-defined architecture: we assume that the sink has a sufficiently powerful power supply to handle the computationally intensive clustering algorithm in an offline phase. Then, it simply transmits the cluster formation (which node belongs to which cluster), the transmission path, and the routing schedule back to the network. Indeed, this approach does not reduce the complexity, but it shifts the complex and heavy calculations away from the resource-constrained sensor nodes.
- Network partitioning and parallel computing: Before running the ARI-DPSO on a network of thousands of nodes directly, we could apply a grid-based approach in which we divide the dense network into local grids, then execute ARI-DPSO through a multi-core edge server to tackle the issue of complexity.

### 3.9. Integrated Algorithm

In ARI-DPSO, the position of a particle $X^{\left(p\right)}$ is represented by a complete cluster configuration vector that encodes nodes to cluster membership. The core objective is to maximize the network’s lifetime ($L^{⋆}$), which is the result of selecting the most energy-efficient particle through the course of the total iterations. The ARI-DPSO process starts with an initialization phase in (lines:1–9) in Algorithm 3. In this phase, the starting position $X^{\left(p\right)}$, the initial personal best solution $P_{\mathrm{best}}$, and the initial global solution $g_{\mathrm{best}}$ are constructed using the method explained in Section 3.2.1 and fed to the ARI-DPSO. Algorithm 1 is invoked at each iteration to construct the data routing paths, calculate the energy load, and compute the fitness (line:4).

The main logic of the swarm’s search is executed in the main iteration loop (lines:10–36) of a maximum number of iterations, $T_{\max}$. In this main loop, we calculate the level of stagnation of each particle by computing its similarity to the global best solution by applying the ARI metric. The ARI score enables us to calculate the coefficient of perturbation $c_{3}$ (lines:13–14). The values of $c_{3}$ are used to repulse the particle away from the global best to avoid stagnation. The calculation of $c_{3}$ invokes the procedure explained in Algorithm 2.
**Algorithm 3** Swarm-based clustering optimization with adaptive perturbation.**Require:** Initial assignments ${\left\{a_{0}^{\left(p\right)}\right\}}_{p=1}^{P}$, Iterations $T_{\max}$, Parameters: $\left(\omega ,c_{1},c_{2},C_{\mathrm{cap}}\right)$**Ensure:** Best cluster assignment $g_{\mathrm{best}}$
▹ Phase 1: Initialization
  1:**for** p=1 to *P* **do**  2:   $X_{0}^{\left(p\right)}\leftarrow a_{0}^{\left(p\right)}$
▹ Set current position from initial assignment (t = 0)
  3:   $V_{p}^{0}\leftarrow \emptyset$
▹ Initialize velocity (t = 0)
  4:   $f_{\mathrm{current}}\leftarrow \mathrm{Fitness}\left(X_{0}^{\left(p\right)}\right)$  5:   $P_{\mathrm{best}}\leftarrow X_{0}^{\left(p\right)}$;  6:   $f_{\mathrm{pBest}}^{\left(p\right)}\leftarrow f_{\mathrm{current}}$  7:**end for**  8:$g_{\mathrm{best}}\leftarrow \arg\max_{P_{\mathrm{best}}}f_{\mathrm{pBest}}^{\left(p\right)}$
▹ Set initial Global Best assignment
  9:$f_{\mathrm{gBest}}\leftarrow \max_{P_{\mathrm{best}}}f_{\mathrm{pBest}}^{\left(p\right)}$
▹ Phase 2: Main Optimization Loop
10:**for** t=0 to $T_{\max}-1$ **do**11:   **for** p=1 to *P* **do**12:   $X_{t}^{\left(p\right)}\leftarrow X^{\left(p\right)}$
▹ Retrieve position from main storage
▹ Compute Adaptive Perturbation Coefficient ($c_{3}$)
13:   $\mathrm{ARI}\leftarrow \mathrm{AdjustedRandIndex}(X_{t}^{\left(p\right)},g_{\mathrm{best}})$14:   $c_{3}\leftarrow C_{\mathrm{cap}}\;\cdot \;\max\left(0,\mathrm{ARI}\right)$
▹ Update velocity and Position
15:   $V_{p}^{t+1}\leftarrow \omega ⊗V_{p}^{t}⊕c_{1}⊗\left(P_{\mathrm{best}}⊖X_{t}^{\left(p\right)}\right)⊕c_{2}⊗\left(g_{\mathrm{best}}⊖X_{t}^{\left(p\right)}\right)⊕c_{3}⊗V_{\mathrm{random}}$16:   $X_{\mathrm{trial}}^{\left(p\right)}\leftarrow X_{t}^{\left(p\right)}⊕V_{p}^{t+1}$17:   $V_{p}^{t}\leftarrow V_{p}^{t+1}$
▹ Store new velocity for next inertia step

▹ Evaluate Trial Position
18:   $X_{t+1}^{\left(p\right)}\leftarrow X_{\mathrm{trial}}^{\left(p\right)}$19:   $f_{\mathrm{current}}\leftarrow \mathrm{Fitness}\left(X_{t+1}^{\left(p\right)}\right)$20:   **if** $f_{\mathrm{current}}>1\times 10^{-9}$ **then**
▹ Validity check for the network connectivity
21:      **if** $f_{\mathrm{current}}>f_{\mathrm{pBest}}^{\left(p\right)}$ **then**22:      $P_{\mathrm{best}}\leftarrow X_{t+1}^{\left(p\right)}$;23:      $f_{\mathrm{pBest}}^{\left(p\right)}\leftarrow f_{\mathrm{current}}$24:      **end if**25:      **if** $f_{\mathrm{current}}>f_{\mathrm{gBest}}$ **then**26:      $g_{\mathrm{best}}\leftarrow X_{t+1}^{\left(p\right)}$;27:      $f_{\mathrm{gBest}}\leftarrow f_{\mathrm{current}}$28:      **end if**29:      $X^{\left(p\right)}\leftarrow X_{t+1}^{\left(p\right)}$
▹ Commit position to main storage
30:   **else**
▹ Move Failed (Non-viable): Hard Reset
31:      $X^{\left(p\right)}\leftarrow \mathrm{GenerateRandomValidAssignment}\left(\right)$32:      $f_{\mathrm{pBest}}^{\left(p\right)}\leftarrow \mathrm{Fitness}\left(X^{\left(p\right)}\right)$;33:      $P_{\mathrm{best}}\leftarrow X^{\left(p\right)}$34:   **end if**35:   **end for**36:**end for**
▹ phase 3: Final Output
37:**return** $g_{\mathrm{best}}$

In ARI-DPSO, the velocity updates: $V_{p}^{t+1}\leftarrow \omega ⊗V_{p}^{t}⊕c_{1}⊗\left(P_{\mathrm{best}}⊖X_{t}^{\left(p\right)}\right)⊕c_{2}⊗\left(g_{\mathrm{best}}⊖X_{t}^{\left(p\right)}\right)⊕c_{3}⊗V_{\mathrm{random}}$ (line:15), are not only dictated by the conventional parameters: Inertia ω, $c_{1}$ and $c_{2}$ but also by the adjusted perturbation parameter $c_{3}\;\cdot \;V_{\mathrm{random}}$. Applying this move to the particle yields the trial assignment $X_{\mathrm{trial}}^{\left(p\right)}$ (line:16). The proposed move is only accepted if it results in a valid cluster configuration with a valid lifetime $f_{\mathrm{current}}>1\times 10^{-9}$ as in (line:20). This check ensures that only particles with connected clusters are accepted into the swarm. The particle’s personal best solution $P_{\mathrm{best}}$ and the swarm’s global best solution $g_{\mathrm{best}}$ are updated in the case where an improved solution is achieved (lines:21–28). Particles that fail to exhibit a valid lifetime are hard-reset into a new random position to try to explore a new search area. This random position is set as the new personal best solution (lines:31–33). The global best clustering configuration $g_{\mathrm{best}}$ that yields the best lifetime $L^{⋆}$ is returned after the preset number of iterations is complete (line:37). The integrated ARI-DPOS approach is summarized in the Algorithm 3, and Table 5 outlines the definition of the terms used in this algorithm.

## 4. Results and Discussion

### 4.1. Simulation Setup

To ensure a fair comparison with the state-of-the-art EBPT-CRA [15], we evaluated the ARI-DPSO and the standard DPSO in a network configuration that mimics the state-of-the-art conditions in terms of network model and deployment, energy consumption model, initial node energy, and number of trials.

#### 4.1.1. Network Model and Deployment

The network consists of N=100 sensor nodes deployed uniformly at random in a sensing area of 300 m×300 m, with the sink located at the center of the sensing area. All sensor nodes are homogeneous and start with an initial energy of $E_{\mathrm{initial}}=1.0\;J$, and all the sensors in this study are static. The sink is assumed to receive control messages over the network and to have a sufficiently powerful power supply to handle the heavy clustering algorithm calculations.

#### 4.1.2. Network Topology and Energy Consumption Model

To establish a network topology in wireless sensor networks, a local neighbor discovery and a global network mapping are required. A method for local neighbor discovery based on path-loss estimation is presented in [35]. Each node sends a beacon message at its maximum transmission power. Neighbor nodes that receive this message measure the received signal strength indicator (RSSI) and deduce the path loss, $L_{\mathrm{path}}$ in decibels (dB), as the difference between the maximum transmission power $P_{\mathrm{tx}}$ and the actual reception power $P_{\mathrm{rx}}$:

(25) $$L_{\mathrm{path}}=P_{\mathrm{tx}}-P_{\mathrm{rx}}.$$

Both nodes recognize each other as neighbors and compute the minimum required transmission energy. The sink uses the flooding protocol [36] to construct a connectivity graph of the entire network. In this protocol, the sink broadcasts flooding messages across the network. Each receiver stores the sender’s ID as a potential parent and transmits the signal to the next node. Once the entire network has been traversed with these flooding messages, nodes can transmit their neighbor lists back to the sink via multi-hop relay nodes.

To evaluate the performance of the ARI-DPSO, we adopted the first-order radio model described in detail in Section 3.1. This radio model is used by the state-of-the-art EBPT-CRA approach, thereby ensuring a fair comparison between the ARI-DPSO and EBPT-CRA.

#### 4.1.3. Statistical Validation

To ensure the validity and robustness of our approach, the results of our simulations are the average of 50 independent trials meant to give a large level of certainty that the achieved performance is not affected by the stochastic nature of the DPSO. The parameters used for this setup are summarized in Table 6.

### 4.2. Performance Evaluation

To test the efficiency of our proposed algorithm, ARI-DPSO, we compared it to a standard DPSO implementation, a genetic algorithm (GA) implementation, an ant colony implementation, and the state-of-the-art EBPT-CRA method [15].

**Figure 2:**
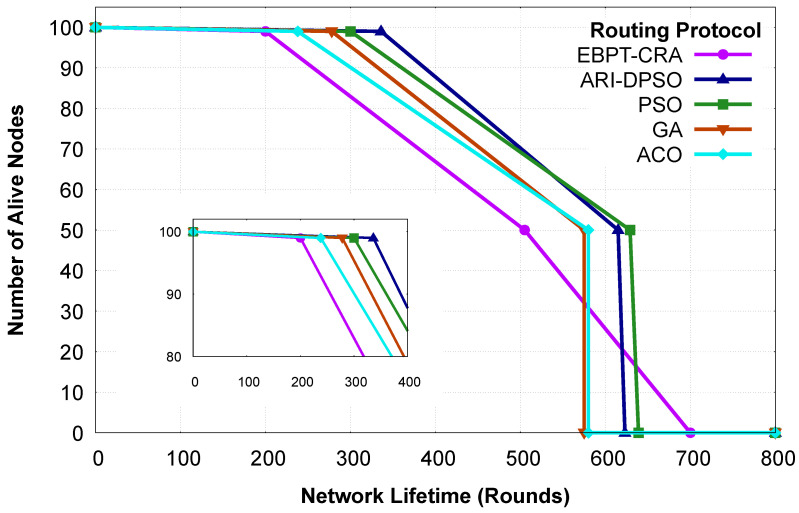
Lifetime progression with an inset highlighting the FND.

These five methods are compared in terms of network longevity, and their performance is shown in Figure 2, which plots the number of dead nodes over the iterations. Our external benchmark EBPT-CRA experiences first node death (FND) at around 200 rounds, and half of the network remains alive for 505 rounds for this method. The ACO experienced FND at 238 rounds, while its HND and LND occurred simultaneously at 580 rounds. Similarly, the GA sustained 278 rounds before the occurrence of FND and had its HND and LND simultaneously at 575 rounds. The standard DPSO exhibits robust performance by delaying the FND until 300 rounds and experiences the half-node death (HND) and last-node death (LND) after 629 and 639 rounds, respectively.

Our proposed method, ARI-DPSO, outperforms the benchmarks in the early and middle stages of the network’s lifetime, securing an FND of 336 rounds. It outperforms the EBPT-CRA by 68 percent and improves on the standard DPSO by 12 percent. It also outperforms both the ACO and GA with 7 percent in terms of HND and 6 percent in terms of LND. Most importantly, it outperforms both approaches by 29 percent and 17 percent, respectively, in terms of FND. The ARI-DPSO method sustains its early stability by delaying the occurrence of the HND until 615 rounds. It is worth noting that the EBPT-CRA method achieves the longest LND among the five methods, with approximately 700 rounds. However, our proposed algorithm prioritizes prolonging the stable period during which the majority of the network’s nodes are operating.

As discussed in Section 3, we built our network mainly to delay its occurrence by defining our fitness function by the death of the first node in the network. The comparison in this section illustrates that the ARI-DPSO not only outperforms the benchmark EBPT-CRA in the first half of the network lifetime, but it also surpasses the standard discrete PSO, which is a simpler version of ARI-DPSO. This points to the vital role played by the perturbation element in securing this supremacy.

### 4.3. The Role of the Perturbation Capacity $C_{\mathrm{cap}}$

The ARI-DPSO relies heavily on the perturbation components in the position and velocity updates of the particle. These components serve as a mechanism to avoid stagnation by repulsing the particles away from the global best solution. However, applying an excessive random perturbation to the swarm could severely destabilize the network.

We conducted a sensitivity analysis to examine the effects of varying the values $C_{\mathrm{cap}}$ on the lifetime of the network and its stability. The number of particles reset reveals the effects of $C_{\mathrm{cap}}$ in the creation of non-viable particles. Thus, it is a suitable test for network stability.

**Figure 3:**
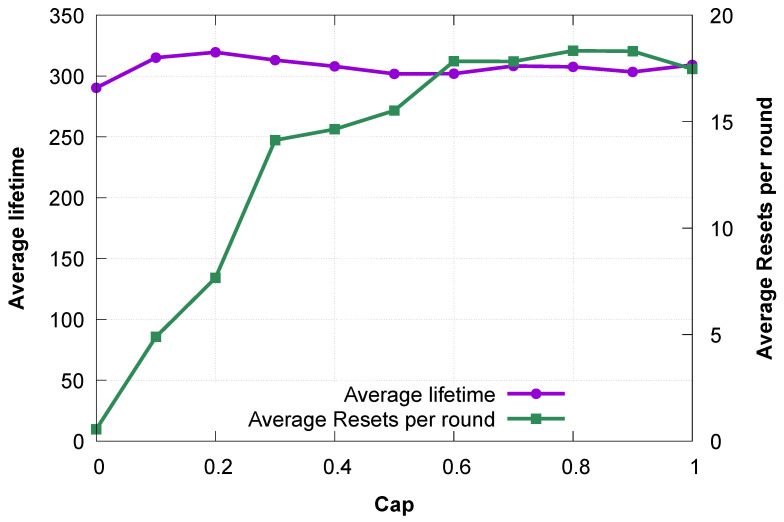
$C_{\mathrm{cap}}$ tuning.

In this sensitivity analysis, we employed 30 particles and 100 iterations to obtain the optimal value of $C_{\mathrm{cap}}$, which achieves network stability and enhances its lifetime. Figure 3 highlights the effects of varying $C_{\mathrm{cap}}$ from 0.0 to 1.0 on the lifetime and the average number of particle resets per round. The optimal lifetime reaches its peak at a $C_{\mathrm{cap}}$ of 0.2, by achieving 319 rounds, while it did not surpass 290 rounds and 315 rounds for $C_{\mathrm{cap}}$ values of 0.0 and 0.1, respectively. At $C_{\mathrm{cap}}$ of 0.0, the effect of the perturbation component $c_{3}$ is deactivated (multiplied by the value of $C_{\mathrm{cap}}$: 0.0). Therefore, the ARI-DPSO at this stage behaves as a standard DPSO, and its average number of particle resets at a $C_{\mathrm{cap}}$ of 0.0 is less than one reset per round. The values of the average particle resets per round are around 5 and 8 resets at $C_{\mathrm{cap}}$ of 0.1 and 0.2, respectively. As the $C_{\mathrm{cap}}$ values increase over 0.2, the swarms start to experience the effect of excessive perturbation, manifesting through a rapid increase in the particle resets that reaches around 15 resets and a drop in the lifetime to reach 301 at $C_{\mathrm{cap}}=0.5$. The lifetime oscillated at the $C_{\mathrm{cap}}$ values of 0.9 and 1.0 to settle to 309 rounds at 1.0, while the average number of particle resets oscillated around 18 resets for both $C_{\mathrm{cap}}$ values.

This analysis reveals that there is a safe zone for the perturbation tuning parameters. $C_{\mathrm{cap}}$ value of 0.2 was a suitable value to inject a sufficient perturbation to propel the particles away from stagnation. It was also moderate to prevent excessive destructive perturbation that might lead to network destabilization. It is relevant to highlight that the ARI-DPSO secured an average lifetime of 336 rounds in Section 4.2 while it did not achieve over 319 rounds in this sensitivity analysis. The reason is that, in this sensitivity analysis, the ARI-DPSO method was tested under 25 trials, completely different from the 50 trials mentioned in Section 4.1, whose results are reported in Section 4.2. The aim is to run the sensitivity analysis on 25 trials to select the value of the optimal $C_{\mathrm{cap}}$, then use this value to run the total experiment on a larger, different input set (the 50 trials).

### 4.4. Swarm Dynamics

The efficacy of any swarm particle search depends heavily on the dynamic movement of this swarm and on its diversity to explore various search areas. To examine the effects of the movement and diversity on the DPSO variant approaches, the standard DPSO and the ARI-DPSO, we will study their diversity and movement over 100 iterations. To weigh the measure of diversity between particles over the course of iterations, we use the values of the 1-ARI measure, which is an ARI-derived dissimilarity measure, to contrast the efficiency of the search behavior of the DPSO approach and the ARI-DPSO. We also record the trajectory of the global best lifetime for both approaches and the number of particle resets.

**Figure 4:**
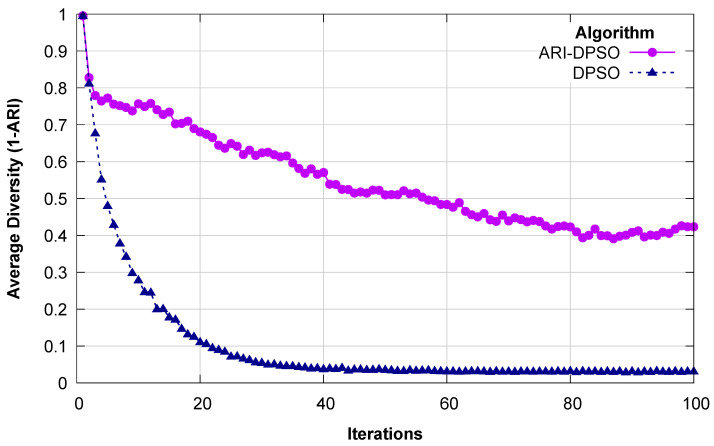
Average diversity per iteration.

**Figure 5:**
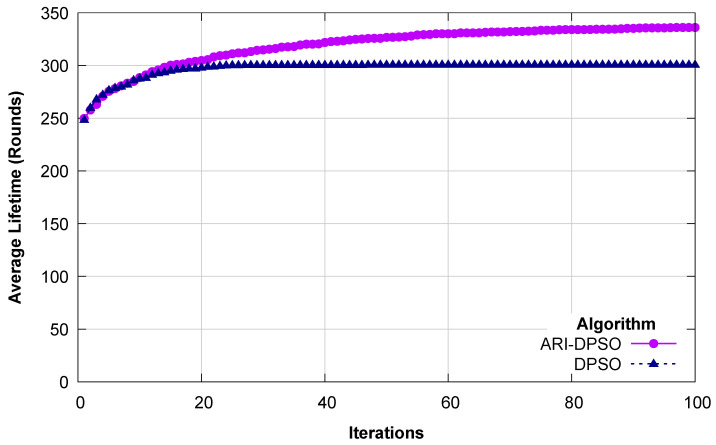
Average lifetime per iteration.

**Figure 6:**
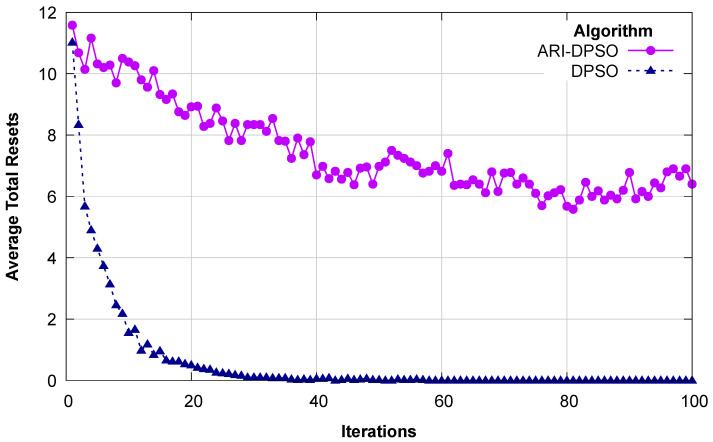
Average number of resets per iteration.

The diversity, the trajectory of the best lifetime, and the average number of resets at iteration in both methods are depicted in Figure 4, Figure 5 and Figure 6.

Figure 4 shows that both approaches start with a high diversity, but the DPSO experiences a rapid collapse by reaching a value of 0.1 within 20 iterations and settles near 0.02 for the last 50 iterations. The ARI-DPSO sustains a relatively higher diversity throughout all 100 iterations. Although it experiences a decrease at the beginning of the process, it manages to secure a diversity score above 0.5 for 50 percent of the process and settles around 0.4 for the last 20 percent of the optimization process. These results provide a visual demonstration of the role played by the perturbation component in preventing premature convergence by introducing randomness into particles that exhibit a high level of similarity to the global best solution. The lack of randomness perturbation drove the DPSO to rapid premature convergence.

Figure 5 reveals that the DPSO and the ARI-DPSO started with almost the same lifetime, around 250 rounds at the first iteration. However, the DPSO did not improve after the 20th iteration, where it reached a lifetime of 300 rounds and remained stagnant for the rest of the optimization search. Conversely, the ARI-DPSO continuously and gradually kept improving over the search iteration, enabling it to secure 336 rounds. This continuous improvement is a result of the swarm diversity that enabled the ARI-DPSO to explore new search areas that are inaccessible to the DPSO.

The particle reset, a mechanism triggered when a particle constructs a non-viable cluster configuration, is strongly correlated with swarm diversity. At the beginning of the search, when particles are still chaotic, the DPSO experienced a relatively high number of resets of around 11 resets per round, as seen in Figure 6. However, at the end of the first 20 iterations, when the DPSO starts to stagnate, the number of average resets per round drops to approach zero. The ARI-DPSO maintained a relatively high number of resets per round, ranging from 6 to 11.

The average per-round lifetime trajectory and diversity illustrate the dynamic and search behavior of the DPSO and the ARI-DPSO. This experiment highlights the role of the perturbation parameter in addressing one of the most critical drawbacks of DPSO: premature convergence. This proposed method is proactive, as it targets the problem of particles converging on the global best and injects a random component to resolve this complication at its source. It also applies the ARI metric, a powerful tool for quantifying the similarity and applying the proportional perturbation needed to alleviate the effects of possible premature convergence.

The relatively high rate of resets, if it is kept within reasonable limits, is an indicator of the successful and dynamic nature of the particle in the ARI-DPSO, as it reveals the swarm’s continuous exploration of new search areas. The improved performance rewards this attempt, while the reset count is a price the swarm pays for its exploration.

### 4.5. Scalability

**Figure 7:**
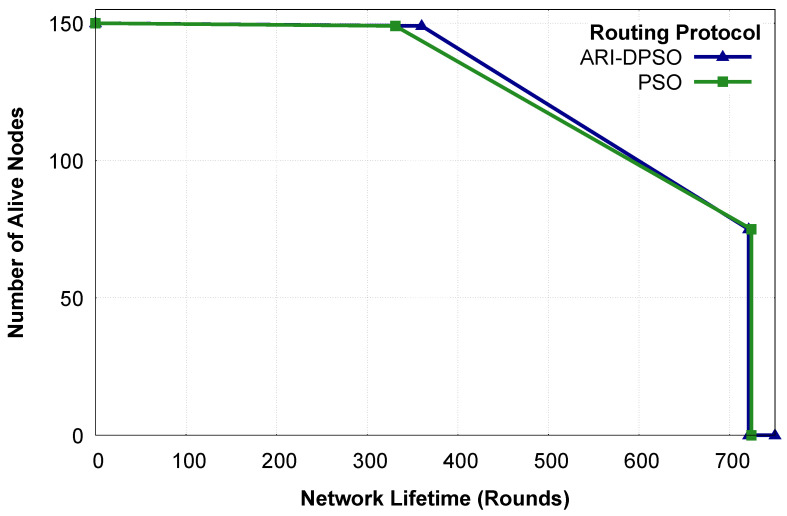
Lifetime progression for 150 nodes.

**Figure 8:**
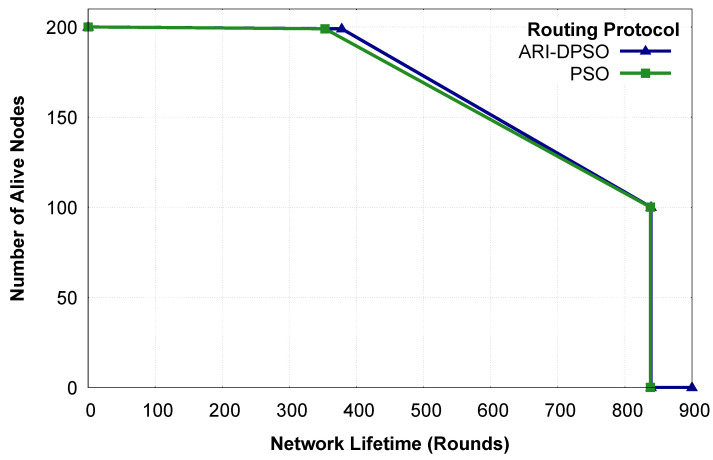
Lifetime progression for 200 nodes.

To test the scalability of ARI-DPSO, we executed it on larger networks: a network of 150 nodes and a network of 200 nodes. Figure 7 and Figure 8 show the lifetime progression of ARI-DPSO and the standard PSO under these dense networks.

In the 150-node network, ARI-DPSO secured a lifetime of 360 rounds before the FND occurrence and has both HND and LND at 721 rounds. Similarly, the standard PSO experienced FND at 331 rounds; its HND and LND occurred at 724 rounds for the same network. For the 200-node networks, ARI-DPSO secured 378 rounds before the occurrence of its FND, while the standard PSO did not surpass 353 rounds. In this network, both the ARI-DPSO and the standard PSO have their LND and HND at 839 rounds. The results above show that ARI-DPSO has around a 7 percent improvement in terms of FND over PSO for 150-node networks and maintains almost the same ratio of improvement for 200-node networks. This shows that ARI-DPSO is capable of maintaining the proportional performance for dense networks, which indicates that ARI-DPSO is scalable to dense networks.

## 5. Conclusions

In this study, we presented the adjusted Rand index-guided DPSO algorithm (ARI-DPSO) to maximize the longevity of wireless sensor networks. The key step to achieving this goal is the formation of a balanced clustering configuration and the minimum energy routing paths. However, we established through comparative analysis of the performance of the ARI-DPSO and the standard DPSO that the premature convergence impedes the latter from reaching new search areas that might contain optimal solutions.

Unlike the standard DPSO, the ARI-DPSO approach employs an adaptive measure: the adjusted Rand index, which acts as an early-stage detection mechanism of the premature convergence. This mechanism proactively detects the likelihood of premature convergence by perpetually comparing the level of similarity between the global best solution and the individual particles in the swarm. Accordingly, it triggers a dynamic perturbation coefficient $c_{3}$. This perturbation parameter, in turn, enhances the swarm search behavior by ensuring that individual particles are driven away from a rapid convergence to the global best solution.

Results from our extensive simulations confirm the superiority of the ARI-DPSO approach over the state-of-the-art EBPT-CRA approach and the standard DPSO algorithm. This superiority is a result of the enhanced swarm search behavior in the ARI-DPSO, which turns the clustering problem into a dynamic and diversity-aware search.

In future work, the ARI-DPSO approach has promising applications in more complex wireless sensor network scenarios, such as those requiring a multi-objective function that optimizes multiple network parameters.

## Figures and Tables

**Table 2:** Node–cluster membership.

Node ID	Current Particle ($X^{\left(p\right)}$)	Global Best ($g_{\mathrm{best}}$)
*c*	*X*	*P*
*d*	*X*	*Q*
*e*	*Y*	*Q*
*f*	*Y*	*Q*
*g*	*Z*	*R*
*h*	*Z*	*R*

**Table 3:** Contingency table.

$X^{\left(p\right)}\setminus g_{\mathrm{best}}$	*P*	*Q*	*R*	Row Sum ($a_{i}$)
*X*	$n_{X,P}=1$	$n_{X,Q}=1$	$n_{X,R}=0$	$a_{X}=2$
*Y*	$n_{Y,P}=0$	$n_{Y,Q}=2$	$n_{Y,R}=0$	$a_{Y}=2$
*Z*	$n_{Z,P}=0$	$n_{Z,Q}=0$	$n_{Z,R}=2$	$a_{Z}=2$
Column Sum ($b_{j}$)	$b_{P}=1$	$b_{Q}=3$	$b_{R}=2$	N=6

**Table 4:** Definition of the terms used for the calculation of ARI.

Term	Symbol(s)	Explanation
Contingency Matrix	*M*	The intermediate table used to organize the raw node assignments. Its cells ($n_{ij}$) count the nodes common to cluster *i* ($X^{\left(p\right)}$ and cluster *j* ($g_{\mathrm{best}}$.
Contingency count	$n_{ij}$	The raw count of nodes that ended up in cluster *i* in $X^{\left(p\right)}$ and in cluster *j* in $g_{\mathrm{best}}$ (a single cell entry i the matrix *M*).
Index (Observed Agreement)	∑i,jnij2	The total number of pairs of nodes that agree by being placed in the same cluster in both solutions.
Cluster Marginal Sums	$a_{i}$ and $b_{j}$	$a_{i}$ is the total number of nodes in cluster *i* of $X^{\left(p\right)}$ (row sum). $b_{j}$ is the total number of nodes in cluster *j* of $g_{\mathrm{best}}$ (column sum).
Total Node Pairs	PN=N2	The total number of unique, unordered pairs of nodes in the network (*N* is the total number of nodes).
Row Pairs Sum	Pa=∑iai2	The total number of pairs placed in the same cluster within the first solution ($X^{\left(p\right)}$), derived from the row marginal sums.
Column Pairs Sum	Pb=∑jbj2	The total number of pairs placed in the same cluster within the second solution $g_{\mathrm{best}}$, derived from the column marginal sums.
Max Index (Maximum Possible Agreement)	Max Index	The theoretical maximum agreement possible given the fixed cluster sizes ($a_{i}$ and $b_{j}$) of the two solutions.
Expected Index (Agreement by chance)	Expected Index	The total number of node pairs expected to agree purely by random chance, assuming the fixed cluster sizes. This is used to correct the ARI metric.

**Table 5:** Comprehensive notations of the discrete PSO algorithm.

Context	Symbol	Meaning
DPSO variables & Inputs (Discrete Adaptive PSO Core Loop)		
Swarm Size	*P*	Total number of particles (solutions) in the swarm.
Maximum Iterations	$T_{\max}$	The maximum number of iterations for the optimization loop.
Swarm Initialization	${\left\{a_{0}^{\left(p\right)}\right\}}_{p=1}^{P}$	set of initial assignment vectors (positions) for *P* particles (used to set $X_{0}^{\left(p\right)}$).
Initial Position	$X_{0}^{\left(p\right)}$	The initial cluster assignment vector (position) of particle *p* at t=0.
Current Position	$X_{t}^{\left(p\right)}$	The cluster assignment vector of particle *p* at the beginning of iteration *t*.
Trial Position	$X_{\mathrm{trial}}^{\left(p\right)}$	The new cluster assignment vector generated by the velocity update before commitment.
Personal Best	$P_{\mathrm{best}}$	The best position found so far by particle *p*.
Global Best	$g_{\mathrm{best}}$	The best position found across the entire swarm.
Velocity (Previous)	$V_{p}^{t}$	The discrete velocity vector (list of moves) used for the current inertia calculation.
Velocity next	$V_{p}^{t+1}$	The new velocity vector calculated in iteration *t*.
Coefficients & Parameters		
Inertia weight	ω	The influence of the previous velocity.
Cognitive Coeff.	$c_{1}$	weight for the motion towards the particle’s own best position ($P_{\mathrm{best}}$).
Social Coeff.	$c_{2}$	weight for the motion towards global best position ($g_{\mathrm{best}}$).
Random Vector	$V_{\mathrm{random}}$	A random discrete vector used for perturbation.
Perturbation Cap	$C_{\mathrm{cap}}$	The maximum possible value of the adaptive perturbation coefficient.
Adaptive Coeff.	$c_{3}$	The calculated adaptive perturbation factor, proportional to the particle’s similarity to $g_{\mathrm{best}}$.
Fitness & Evaluation		
Current Fitness	$f_{\mathrm{current}}$	The WSN Lifetime calculated for the current position ($X_{t+1}^{\left(p\right)}$).
pBest Fitness	$f_{\mathrm{pBest}}^{\left(p\right)}$	The WSN Lifetime achieved by the personal best position $P_{\mathrm{best}}$.
gBest Fitness	$f_{\mathrm{gBest}}$	The maximum WSN Lifetime achieved by the global best assignment $g_{\mathrm{best}}$.
Max Lifetime	$L^{⋆}$	The maximum WSN Lifetime (fitness) achieved by the $g_{\mathrm{best}}$ assignment.

**Table 6:** Simulation and optimization parameters.

Parameter Description	Symbol	Value
A. Network and Energy Model		
Deployment Area Size	D×D	300 m×300 m
Number of Nodes	*N*	100
Base Station (BS) Location	$\left(x_{\mathrm{BS}},y_{\mathrm{BS}}\right)$	(150,150)
Initial Node Energy	$E_{\mathrm{initial}}$	1.0 J
Data Packet Length	*l*	2500 bit
Data Generation Rate	$R_{\mathrm{data}}$	2500 bit/round
Electronic Processing Energy	$E_{\mathrm{elec}}$	50 nJ/bit
Free Space Coeff.	$\epsilon_{\mathrm{fs}}$	$10\;\mathrm{pJ}/\mathrm{bit}\;\cdot \;m^{2}$
B. DPSO Optimization Parameters		
Optimization Iterations	$T_{\max}$	100
Swarm Size	$P_{\mathrm{particles}}$	30
Standard DPSO Inertia	$\omega_{\mathrm{std}}$	0.7 (Fixed)
Standard DPSO Cognitive Coeff.	$c_{1,\mathrm{std}}$	0.8 (Fixed)
Standard DPSO Social Coeff.	$c_{2,\mathrm{std}}$	0.8 (Fixed)
ARI-DPSO Perturbation cap	$C_{\mathrm{cap}}$	0.2 (Tuned)

## Data Availability

The data presented in this study is available on request from the corresponding author.
